# Trends in Acuity of Residents in Assisted Living

**DOI:** 10.1093/geroni/igab046.2030

**Published:** 2021-12-17

**Authors:** Cassandra Hua

**Affiliations:** Brown University, Providence, Rhode Island, United States

## Abstract

Assisted living serves as a substitute for nursing home residents with low care needs, especially in markets with a high proportion of dually eligible Medicare beneficiaries. This study examines trends in the acuity of residents in assisted living communities over time in comparison to nursing homes to characterize how substitution has affected the resident compositions of both settings. We also examine how trends in acuity are shaped by dual eligibility. Using Medicare claims data, we identify cross-sectional samples of beneficiaries in each setting from 2007-2017. The proportion of residents in assisted living with high care needs has increased 18% in assisted living communities compared to 8.7% in nursing homes. Acuity levels are higher among dually eligible assisted living residents compared to assisted living residents who are not dually eligible. Policy makers and administrators should examine whether assisted living is prepared to provide care for an increasingly acute population.

